# Development and validation of a nomogram for early prediction of sepsis-associated liver injury: a multicenter study

**DOI:** 10.3389/fmed.2026.1899424

**Published:** 2026-08-21

**Authors:** Ruimin Tan, Shuwei Zhang, Quansheng Du, Jingmei Wang, Donglin Fu, Yunxing Cao

**Affiliations:** 1 Department of Critical Care, Chongqing Academy of Medical Sciences, Chongqing General Hospital, Chongqing University, Chongqing, China; 2 Department of Emergency Medicine and Critical Care Medicine, Hebei General Hospital, Shijiazhuang, Hebei, China; 3 Critical Care Department, Handan Central Hospital, Handan, Hebei, China

**Keywords:** ICU, model development and validation, multicenter study, nomogram, sepsis-associated liver injury

## Abstract

**Background:**

Sepsis-associated liver injury (SALI) is an important manifestation of sepsis-associated organ dysfunction and is associated with adverse clinical outcomes. However, practical tools for estimating the risk of subsequent SALI in critically ill patients with sepsis remain limited. This study aimed to develop and geographically validate a nomogram based on routinely available clinical variables using multicenter retrospective data.

**Methods:**

Sepsis patients admitted to the ICUs of Hebei General Hospital and Handan Central Hospital, East District, between March 1, 2021, and June 1, 2024, were retrospectively enrolled. A total of 847 eligible patients from these two centers constituted the model development and internal validation cohort and were randomly divided into a training cohort and a testing cohort at a 6:4 ratio. An independent cohort of 424 sepsis patients admitted to the ICU of Chongqing General Hospital between March 1, 2021, and January 1, 2026, was included for external geographic validation. Demographic characteristics, comorbidities, laboratory parameters, and clinical interventions were collected. Candidate predictors were initially screened using univariable analysis and least absolute shrinkage and selection operator (LASSO) regression, followed by multivariable logistic regression to derive the final prediction model for subsequent in-hospital SALI. A nomogram was constructed using the rms package. Model discrimination was assessed using receiver operating characteristic (ROC) curves and the area under the curve (AUC), calibration was evaluated using calibration plots and quantitative calibration metrics, and potential decision-analytic value was assessed using decision curve analysis (DCA).

**Results:**

A total of 1,271 patients with sepsis from three centers were included in the final analysis. The development and internal validation cohort comprised 847 patients, of whom 352 developed SALI (41.6%), and was randomly divided into a training cohort of 508 patients and a testing cohort of 339 patients. The independent external geographic validation cohort comprised 424 patients, of whom 183 patients developed SALI (43.2%). After feature selection and multivariable analysis, age, direct bilirubin, aspartate aminotransferase, prothrombin time–international normalized ratio, and vasoactive-drug use were retained as predictors associated with subsequent in-hospital SALI. The nomogram demonstrated favorable discrimination, with AUCs of 0.925 (95% CI, 0.901–0.948), 0.918 (95% CI, 0.888–0.948), and 0.884 (95% CI, 0.849–0.918) in the training, testing, and external geographic validation cohorts, respectively. Calibration was close to the ideal values in the training cohort but deteriorated in the testing and external geographic validation cohorts, particularly in the external cohort, in which the calibration-in-the-large was 0.945 and the calibration slope was 0.600. Decision curve analysis suggested potential net benefit within an illustrative threshold-probability range of 0.10–0.40.

**Conclusion:**

The nomogram demonstrated favorable discrimination across the training, testing, and external geographic validation cohorts, although calibration deteriorated outside the training cohort. The model may provide a practical approach for estimating subsequent in-hospital SALI risk and identifying patients who may warrant closer clinical assessment. However, local recalibration, prospective validation, and clinical-impact evaluation are required before application in new clinical settings. This retrospective prediction study does not establish that implementation of the nomogram guides effective interventions, improves patient prognosis, or optimizes ICU resource allocation.

## 1 Introduction

Sepsis is a life-threatening organ dysfunction caused by a dysregulated host response to infection (1). Sepsis-associated liver injury (SALI) is an important manifestation of sepsis-associated organ dysfunction and may present as cholestasis, hepatocellular injury, or impaired hepatic synthetic function (2). Previous studies have reported liver dysfunction in approximately 20–50% of critically ill patients with sepsis, and the development of clinically significant hepatic dysfunction is associated with an increased risk of adverse outcomes (3, 4). Because the liver plays central roles in immune regulation, metabolism, and detoxification, hepatic dysfunction may further contribute to systemic inflammation and multiple-organ dysfunction (5). Early identification of patients at increased risk of SALI may support closer clinical assessment and risk-stratified monitoring; however, whether model-guided risk stratification improves clinical outcomes requires prospective evaluation.

The pathophysiology of SALI is complex and involves dysregulated inflammation, impaired hepatic microcirculation, altered immunometabolism, oxidative stress, and mitochondrial dysfunction (6, 7). Excessive production of inflammatory mediators, including tumor necrosis factor-*α* and interleukins, may contribute to hepatocellular injury and hepatic sinusoidal endothelial dysfunction. In parallel, impaired hepatic perfusion and microcirculatory disturbances may promote tissue hypoxia and disordered energy metabolism (8, 9). Sepsis-associated cholestasis is another common manifestation of liver dysfunction and is characterized by impaired bilirubin metabolism and hepatobiliary transport (10). These interacting processes may contribute to progressive hepatic dysfunction and adverse outcomes during sepsis.

A nomogram translates a regression model into a graphical format for individual risk estimation and may facilitate clinical interpretation when it is based on routinely available variables (11). Nomogram-based prediction models have been applied in several critical-care settings, including the prediction of sepsis-associated organ dysfunction (12, 13).

We therefore developed and geographically validated a nomogram based on routinely available clinical variables to estimate the risk of subsequent in-hospital SALI in patients with sepsis. The model was intended to support individual risk estimation and early risk stratification rather than to replace clinical assessment or establish a specific treatment strategy.

## 2 Materials and methods

### 2.1 Data source

This was a multicenter retrospective cohort study. Patients with sepsis admitted to the ICUs of Hebei General Hospital and Handan Central Hospital, East District, between March 1, 2021, and June 1, 2024, were retrospectively screened using the same prespecified eligibility and complete-case criteria. Eligible patients from these two hospitals were pooled to form the development and internal-validation cohort and were subsequently randomly divided into the training and testing cohorts at a 6:4 ratio. After the two development-center datasets were pooled, center-specific SALI outcome status, baseline summaries, and outcome-timing information were not retained separately and could not be reliably reconstructed. Consequently, formal development-center–specific comparisons and leave-one-center-out validation could not be performed using the retained pooled analytical dataset.

Patients with sepsis admitted to the ICU of Chongqing General Hospital between March 1, 2021, and January 1, 2026, were screened using the same inclusion, exclusion, predictor, outcome, and complete-case criteria and were reserved exclusively for external geographic validation. Because the recruitment period at the validation center overlapped with that of the development centers, this assessment represents geographic rather than strictly temporal validation. The same eligibility criteria, definition of T0, SALI criteria, predictor-assessment window, outcome-ascertainment rules, complete-case requirements, variable definitions, measurement units, and coding procedures were applied across all three centers. Laboratory testing was performed using routine local platforms, and local reference intervals and routine ICU treatment protocols were not formally harmonized. The same fixed absolute SALI thresholds and the same operational definition of vasoactive-drug use were applied across centers.

The study was approved by the Ethics Committees of Hebei General Hospital (No. 2026-LW-194), Handan Central Hospital (No. 2026058), and Chongqing General Hospital (No. KY S2024-037-01). Because of the retrospective design and use of routinely collected clinical data, the requirement for written informed consent was waived by the respective ethics committees.

### 2.2 Study population

This study included adult patients who fulfilled the Sepsis-3 diagnostic criteria and were admitted to the ICU for the first time (1). T0 was defined as the index time at which a patient first fulfilled the Sepsis-3 criteria and baseline clinical assessment was initiated.

**Figure 1:**
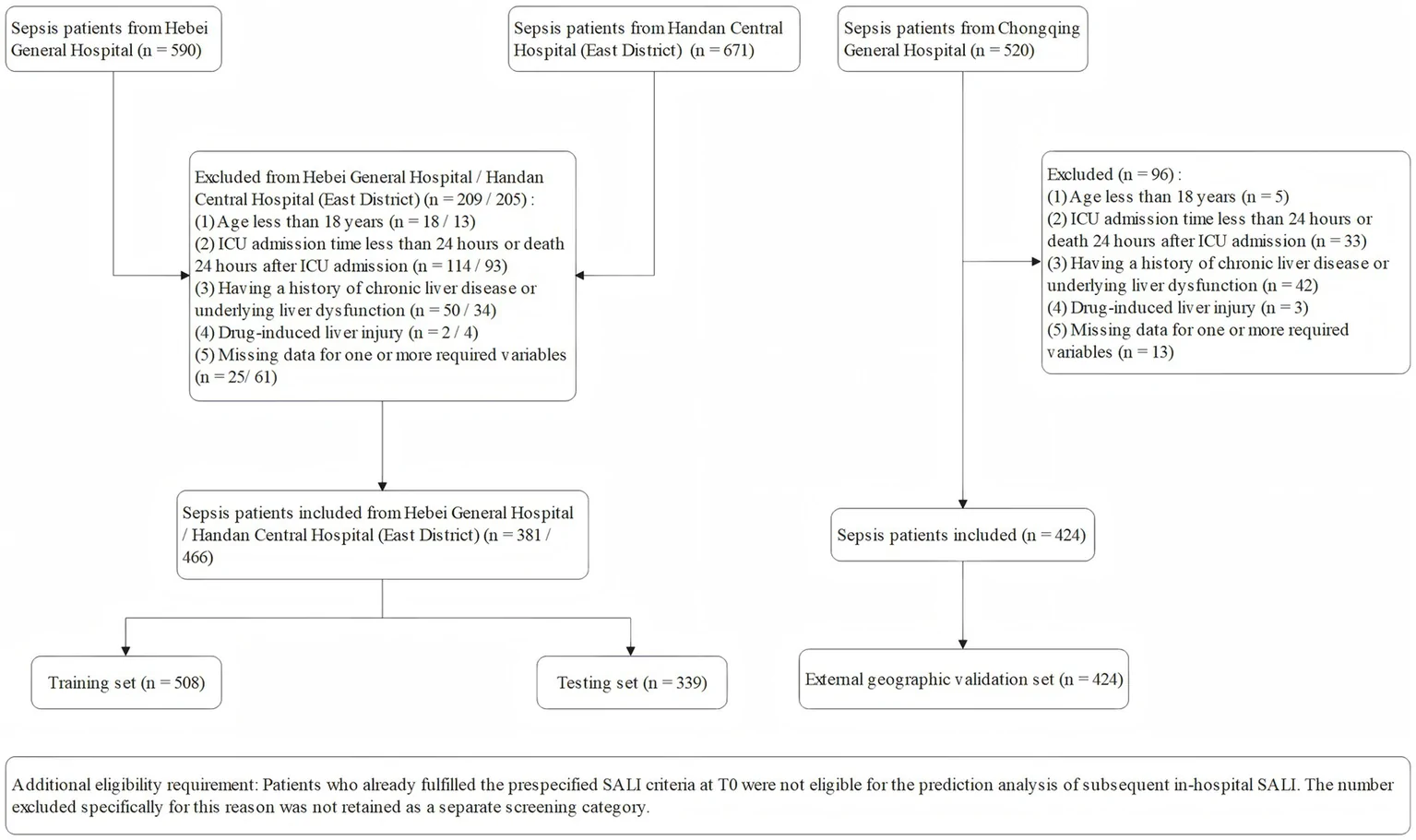
Patient screening and cohort-construction flow. All 1,271 patients included in the final analytical cohorts were free of SALI at T0, and all analyzed SALI events occurred subsequently during hospitalization. Required variables included all prespecified candidate predictors and ALT and TBIL, which were required for SALI outcome ascertainment. The number of patients excluded during initial screening specifically because SALI was already present at T0 was not retained as a separate exclusion category. SALI, sepsis-associated liver injury; ICU, intensive care unit; ALT, alanine aminotransferase; TBIL, total bilirubin.

The exclusion criteria were as follows: (1) age <18 years; (2) ICU stay <24 h or death within 24 h after ICU admission; (3) pre-existing chronic liver disease or baseline hepatic dysfunction, including chronic viral hepatitis, liver cirrhosis, malignant liver tumors, alcoholic liver disease, or a history of liver-related surgery; (4) confirmed drug-induced liver injury; and (5) missing data for any prespecified candidate predictor or for ALT or TBIL, which were required for SALI outcome ascertainment. The detailed patient-selection process is presented in Figure 1.

Patients were eligible for the analytical cohort only if they had not fulfilled the prespecified SALI criteria at T0. Accordingly, patients with SALI already present at the index time were not eligible for the prediction analysis of subsequent in-hospital SALI.

SALI was operationally defined as the first occurrence after T0 of TBIL >34.2 μmol/L and/or ALT >80 U/L in patients with sepsis. This prespecified composite biochemical outcome was adopted from a previous SALI prediction study that applied the same fixed thresholds and was not derived from the present dataset (12). The adult expert consensus on acute liver injury provides contextual support for the ALT threshold but does not establish the entire composite definition as a universally accepted SALI criterion (14). The same fixed absolute thresholds were applied across all three participating hospitals. Laboratory testing was performed using routine local platforms, and local reference intervals were not formally harmonized. Center-specific reference limits were not retained in the analytical dataset and could not be reliably reconstructed; therefore, outcome classification was based on the same absolute thresholds rather than hospital-specific multiples of the upper limit of normal. SALI onset was defined as the time of the first laboratory measurement after T0 that met either criterion. TBIL and ALT were used exclusively for outcome ascertainment and were not included as predictors in the final nomogram. Patients who did not develop SALI were followed for outcome ascertainment until hospital discharge.

### 2.3 Data extraction

Clinical data were extracted from the electronic medical record systems of the three participating hospitals. The collected variables included: (1) demographic characteristics, including age and sex; (2) comorbidities, including coronary heart disease, hypertension, diabetes mellitus, chronic kidney disease, cerebrovascular disease, chronic obstructive pulmonary disease, malignancy, and recent surgery; (3) primary infection sites, including pulmonary, urinary-tract, bloodstream, abdominal, central-nervous-system, skin and soft-tissue, and other infections; (4) illness-severity scores, including the Sequential Organ Failure Assessment (SOFA) score and Acute Physiology and Chronic Health Evaluation II (APACHE II) score; (5) vital signs, including body temperature, heart rate, systolic blood pressure, diastolic blood pressure, and mean arterial pressure; (6) laboratory measurements, including blood-gas parameters, liver- and kidney-function indices, procalcitonin, complete blood-count indices, electrolytes, and coagulation parameters; (7) clinical interventions, including vasoactive-drug use, continuous renal replacement therapy, central venous catheterization, mechanical ventilation, anticoagulant use, glucocorticoid use, and blood-product transfusion; and (8) ICU and hospital lengths of stay.

Baseline laboratory values and vital signs were obtained from the earliest eligible measurement recorded at T0 or within 24 h after T0. For patients who subsequently developed SALI, the predictor-assessment window ended at the time of SALI onset. Therefore, only measurements recorded before the first laboratory result fulfilling the SALI criteria were eligible for predictor extraction. If multiple eligible measurements were available, the earliest measurement was used. Laboratory results, vital signs, and treatment information recorded at or after SALI onset were excluded from the analysis.

Vasoactive-drug use was coded as present when at least one vasoactive agent was administered between T0 and the earlier of 24 h after T0 or SALI onset. Vasoactive-drug administration occurring only at or after SALI onset was not coded as a predictor. For patients who developed SALI within 24 h after T0, the predictor-assessment window was truncated immediately before SALI onset. The same prespecified vasoactive-agent list and coding rule were applied consistently across all three participating centers, as detailed in Supplementary Table S3. The temporal framework for predictor ascertainment and outcome assessment is illustrated in Supplementary Figure S4.

### 2.4 Missing-data handling

A complete-case analysis was performed. Patients with a missing value for any prespecified candidate predictor or for ALT or TBIL, which were required for SALI outcome ascertainment, were excluded before model development and validation. No missing-value imputation was performed. Consequently, the final analytical cohorts contained no missing values for the variables included in the analysis.

### 2.5 Statistical analysis

The distributions of continuous variables were assessed using the Shapiro–Wilk test. Normally distributed variables are presented as mean ± standard deviation and were compared using the independent-samples t test. Non-normally distributed variables are presented as median (25th–75th percentile) and were compared using the Mann–Whitney U test. Categorical variables are presented as *n* (%) and were compared using the chi-square test or Fisher’s exact test, as appropriate.

Candidate predictors were specified before model fitting based on clinical relevance, previous studies of sepsis-associated organ dysfunction, and routine availability during early ICU management. Continuous variables were entered on their original measurement scales, and categorical variables were coded as binary indicators. Variables with *p* < 0.05 in the exploratory univariable analysis were entered into least absolute shrinkage and selection operator (LASSO) regression. Ten-fold cross-validation was used to select the regularization parameter corresponding to the minimum cross-validated error (*λ*_min = 0.006894539). Predictors with nonzero coefficients at the selected λ were subsequently entered into forward stepwise multivariable logistic regression, with entry and removal criteria of *α* = 0.05 and α = 0.10, respectively, to derive the final prediction model. A nomogram was then constructed from the final logistic regression model using the rms package in R.

Model discrimination was assessed using receiver operating characteristic (ROC) curves and areas under the curve (AUCs) with 95% confidence intervals. Calibration was initially assessed using calibration plots and the Hosmer–Lemeshow goodness-of-fit test. The calibration plots display observed probabilities on the y-axis and predicted probabilities on the x-axis; the ideal line represents perfect agreement, the apparent line represents performance in the corresponding cohort, and the bias-corrected line was estimated using 1,000 bootstrap resamples. Additional calibration statistics, classification-threshold procedures, and benchmark-model comparisons are described below.

Decision curve analysis (DCA) was performed using the rmda package to evaluate the potential decision-analytic value of the nomogram across a range of threshold probabilities. The “None” strategy assumes that no patient undergoes the model-triggered clinical action and has a net benefit of zero, whereas the “All” strategy assumes that all patients undergo that action. The model was considered to provide potential net benefit at threshold probabilities where its curve lay above both reference strategies. Because the present model was intended to support risk-based surveillance and clinical reassessment rather than mandate a specific treatment, the DCA results were interpreted as decision-analytic evidence rather than proof of improved clinical outcomes.

The functional forms of age, DBIL, AST, and PT-INR were evaluated in the training cohort using restricted cubic splines with three knots placed at the 10th, 50th, and 90th percentiles. Departure from linearity was assessed by testing the nonlinear spline components. The corresponding restricted cubic spline plots are presented in Supplementary Figure S5. Multicollinearity in the final prediction model was evaluated using variance inflation factors, with VIF values <5 considered indicative of no important multicollinearity. Potentially influential observations were assessed using Cook’s distance, with values greater than 4/*n* considered potentially influential. Where potentially influential observations were identified, the final model was refitted after their exclusion as a sensitivity analysis.

Calibration-in-the-large and calibration slope were estimated in each cohort, with ideal values of 0 and 1, respectively. The 95% confidence intervals for the Brier score were obtained using 2,000 nonparametric bootstrap resamples.

A classification cutoff was selected exclusively in the training cohort by maximizing the Youden index. The same fixed cutoff was subsequently applied unchanged to the testing and external geographic validation cohorts. Sensitivity, specificity, positive predictive value, and negative predictive value were reported with 95% confidence intervals estimated using the Wilson score method. The cutoff was considered a data-derived operating threshold for performance description rather than a universally applicable clinical threshold.

To evaluate incremental predictive performance, a benchmark logistic regression model using baseline total SOFA score as the sole predictor was developed in the training cohort and applied unchanged to the testing cohort. The AUCs of the nomogram and SOFA-only model were compared using the DeLong test. Incremental discrimination was further evaluated using the difference in AUC and the integrated discrimination improvement.

SPSS software (version 27.0) and R software (version 4.3.1) were used for all statistical analyses. Statistical significance was defined as a two-sided *p* value less than 0.05.

### 2.6 Reporting guideline

This study was reported in accordance with the Transparent Reporting of a Multivariable Prediction Model for Individual Prognosis or Diagnosis (TRIPOD) guideline for prediction-model development and external validation. A completed TRIPOD checklist is provided as a separate Supplementary File.

## 3 Results

### 3.1 Baseline characteristics

A total of 590 patients with sepsis were screened at Hebei General Hospital, of whom 209 were excluded and 381 were finally included. At Handan Central Hospital, East District, 671 patients were screened, 205 were excluded, and 466 were finally included. Among the excluded patients, 25 patients at Hebei General Hospital and 61 patients at Handan Central Hospital were excluded because of missing one or more required variables. The two development-center cohorts were subsequently pooled, yielding 847 eligible patients. At Chongqing General Hospital, 520 patients were screened, 96 were excluded, and 424 were included in the external geographic validation cohort; 13 patients were excluded because of missing one or more required variables (Figure 1). The final analytical cohorts consisted exclusively of complete cases, and no missing-value imputation was performed.

Overall, 1,271 patients with sepsis were included in the final analysis. The pooled development and internal-validation cohort comprised 847 patients, including 352 patients who subsequently developed in-hospital SALI and 495 patients who did not. This cohort was randomly divided into a training cohort of 508 patients and a testing cohort of 339 patients at a ratio of 6:4. In the training cohort, 211 patients developed SALI (41.5%), compared with 141 patients (41.6%) in the testing cohort. The external geographic validation cohort comprised 424 patients, of whom 183 subsequently developed SALI (43.2%) and 241 did not. The baseline characteristics of the training and testing cohorts are presented in Supplementary Table S1, and those of the external geographic validation cohort are presented in Supplementary Table S2.

Although the final sample sizes were available for each hospital, center-specific SALI outcome status and baseline summaries for the two development hospitals were not retained separately after pooling and could not be reliably reconstructed. The exact interval from T0 to SALI onset was not retained as an analytical variable for any of the three participating hospitals. Consequently, separate SALI incidence estimates, baseline comparisons, and time-to-SALI distributions could not be reported for Hebei General Hospital and Handan Central Hospital, and formal development-center–specific effect analyses could not be performed using the retained pooled dataset. By design, none of the 1,271 patients included in the final analytical cohorts fulfilled the SALI criteria at T0, and all analyzed SALI events occurred subsequently during hospitalization.

Compared with the testing cohort, the training cohort had lower ALT levels, higher PT and PT-INR values, lower proportions of pulmonary and central-nervous-system infections, and lower proportions of patients receiving cryoprecipitate and packed red-blood-cell transfusions (all *p* < 0.05). No statistically significant differences were observed for the remaining characteristics (all *p* > 0.05).

The baseline clinical features of the 508 sepsis patients in the training cohort are compiled in Table 1. Compared with patients without SALI, patients who subsequently developed SALI were younger and had higher body temperatures, lactate, TBIL, DBIL, ALT, AST, serum sodium, blood urea nitrogen, creatinine, procalcitonin, PT, APTT, PT-INR, platelet-large-cell ratio, mean platelet volume, and platelet distribution width. They also more frequently received central venous catheterization, vasoactive drugs, and platelet, plasma, and cryoprecipitate transfusions, but had lower platelet counts and plateletcrit values. No statistically significant between-group differences were observed for the remaining variables (all *p* > 0.05).

**Table 1:** Baseline comparison of SALI and non-SALI in the training set.

Variables	SALI (*n* = 211)	Non-SALI (*n* = 297)	Z/χ^2^/Fisher	*P*
Demographic data				
Male, *n* (%)	140 (66.35%)	177 (59.60%)	χ^2^ = 2.399	0.121
Age, years	71.00 (60.00,80.00)	76.00 (65.00,83.00)	Z = 3.135	0.002
Underlying diseases, *n* (%)				
Coronary heart disease	52 (24.64%)	61 (20.54%)	χ^2^ = 1.202	0.273
Hypertension	88 (41.71%)	133 (44.78%)	χ^2^ = 0.475	0.491
Diabetes	61 (28.91%)	79 (26.60%)	χ^2^ = 0.330	0.566
Chronic obstructive pulmonary disease	31 (14.69%)	39 (13.13%)	χ^2^ = 0.253	0.615
Cerebrovascular disease	70 (33.18%)	118 (39.73%)	χ^2^ = 2.274	0.132
Chronic kidney disease	48 (22.75%)	77 (25.93%)	χ^2^ = 0.671	0.413
Malignancy	28 (13.27%)	40 (13.47%)	χ^2^ = 0.004	0.949
History of surgery within 3 months	100 (47.39%)	145 (48.82%)	χ^2^ = 0.101	0.751
Infection sites, *n* (%)				
Pulmonary	138 (65.40%)	194 (65.32%)	χ^2^ = 0.000	0.985
Abdominal	82 (38.86%)	117 (39.39%)	χ^2^ = 0.015	0.904
Blood	31 (14.69%)	36 (12.12%)	χ^2^ = 0.712	0.399
Urinary system	35 (16.59%)	60 (20.20%)	χ^2^ = 1.060	0.303
Central nervous system	3 (1.42%)	3 (1.01%)	Fisher	0.696
Skin and soft tissue	4 (1.90%)	10 (3.37%)	χ^2^ = 0.996	0.318
Other	10 (4.74%)	9 (3.03%)	χ^2^ = 1.001	0.317
ICU disease severity scores, M (Q₁, Q₃)				
APACHE score	24.00 (18.00,28.00)	23.00 (18.00,28.00)	Z = 0.933	0.351
SOFA score	9.00 (7.00,11.00)	9.00 (7.00,10.00)	Z = 1.700	0.088
Vital signs, M (Q₁, Q₃)				
Temperature (°C)	36.60 (36.00,37.30)	36.50 (36.00,37.00)	Z = 2.677	0.007
HR (times/min)	96.00 (84.00,113.50)	97.00 (83.00,112.00)	Z = 0.721	0.471
SBP (mmHg)	119.00 (102.50,138.00)	123.00 (105.00,141.00)	Z = 1.574	0.116
DBP (mmHg)	67.00 (57.50,76.00)	67.00 (56.00,78.00)	Z = 0.159	0.874
MAP (mmHg)	84.33 (74.00,94.67)	86.00 (75.67,95.00)	Z = 0.868	0.386
RR (times/min)	20.00 (16.00,26.00)	20.00 (15.00,25.00)	Z = 0.918	0.357
Laboratory tests, M (Q₁, Q₃)				
pH	7.37 (7.29,7.44)	7.38 (7.31,7.42)	Z = 0.323	0.747
lactate (mmol/L)	2.96 (1.80,5.30)	2.00 (1.40,3.39)	Z = 5.078	<0.001
AB (mmol/L)	20.60 (16.85,24.70)	21.80 (18.10,24.90)	Z = 1.908	0.056
OI	201.99 (139.10,301.47)	229.00 (163.83,330.00)	Z = 1.879	0.06
TP (g/L)	50.80 (45.25,57.75)	51.10 (44.00,59.90)	Z = 0.416	0.678
ALB (g/L)	28.00 (24.25,31.30)	27.80 (23.80,31.90)	Z = 0.161	0.872
TBIL (μmol/L)	37.30 (22.00,49.50)	14.40 (10.90,19.50)	Z = 13.060	<0.001
DBIL (μmol/L)	14.10 (7.70,30.65)	5.80 (3.70,9.00)	Z = 11.190	<0.001
ALT (U/L)	80.80 (24.00,147.80)	18.00 (12.00,30.30)	Z = 11.203	<0.001
AST (U/L)	88.00 (40.05,226.15)	30.00 (21.00,54.90)	Z = 10.891	<0.001
K (mmol/L)	3.92 (3.50,4.53)	4.00 (3.60,4.60)	Z = 0.680	0.496
Na (mmol/L)	141.00 (136.45,145.00)	139.00 (135.00,144.00)	Z = 2.197	0.028
Cl (mmol/L)	105.00 (101.00,110.65)	105.00 (101.00,110.00)	Z = 0.608	0.544
Ca (mmol/L)	1.98 (1.86,2.15)	2.00 (1.85,2.16)	Z = 0.305	0.760
PO_4_ (mmol/L)	1.04 (0.77,1.42)	1.12 (0.83,1.38)	Z = 1.022	0.307
Mg (mmol/L)	0.84 (0.71,0.94)	0.81 (0.70,0.95)	Z = 0.254	0.800
BUN (mmol/L)	14.03 (8.84,21.83)	11.70 (7.50,19.30)	Z = 2.533	0.011
Cr (μmol/L)	125.30 (84.40,203.70)	108.10 (70.10,164.70)	Z = 3.230	0.001
PCT (ng/mL)	17.54 (3.10,60.05)	6.66 (1.88,26.67)	Z = 4.496	<0.001
PT (s)	17.30 (14.65,19.70)	14.40 (12.90,15.80)	Z = 9.574	<0.001
PT-INR	1.52 (1.31,1.70)	1.27 (1.15,1.42)	Z = 9.660	<0.001
APTT (s)	36.70 (32.00,44.65)	34.20 (29.90,39.20)	Z = 3.767	<0.001
FIB (g/L)	5.14 (3.48,11.90)	5.65 (3.66,9.27)	Z = 0.733	0.464
TT (s)	16.00 (6.54,17.25)	15.60 (13.70,17.10)	Z = 0.019	0.985
WBC (10^9^/L)	12.66 (7.76,19.20)	11.34 (6.93,17.08)	Z = 1.756	0.079
NEU (10^9^/L)	11.57 (6.39,17.12)	10.15 (5.85,15.28)	Z = 1.801	0.072
LYM (10^9^/L)	0.59 (0.36,0.98)	0.69 (0.41,1.03)	Z = 1.673	0.094
MON (10^9^/L)	0.41 (0.18,0.64)	0.39 (0.20,0.65)	Z = 0.148	0.883
Hb (g/L)	107.00 (92.00,127.00)	104.00 (86.00,124.00)	Z = 1.592	0.111
RDW (fL)	49.00 (45.10,55.00)	48.30 (45.00,54.00)	Z = 0.829	0.407
PLT (10^9^/L)	117.00 (68.50,150.50)	163.00 (97.00,241.00)	Z = 5.335	<0.001
PDW	15.50 (12.90,17.05)	14.00 (11.90,16.30)	Z = 3.262	0.001
Plateletcrit	0.13 (0.08,0.19)	0.19 (0.12,0.26)	Z = 5.052	<0.001
P-LCR	11.47 ± 1.40	11.12 ± 1.43	t = −2.710	0.007
MPV (fL)	36.10 (28.30,43.05)	33.10 (25.60,40.40)	Z = 2.967	0.003
Interventions, *n* (%)				
Deep venous catheterization	174 (82.46%)	223 (75.08%)	χ^2^ = 3.935	0.047
Anticoagulant drugs	102 (48.34%)	156 (52.53%)	χ^2^ = 0.864	0.353
Mechanical ventilation	187 (88.63%)	260 (87.54%)	χ^2^ = 0.137	0.711
Hormones	160 (75.83%)	220 (74.07%)	χ^2^ = 0.202	0.653
Vasoactive drugs	206 (97.63%)	274 (92.26%)	χ^2^ = 6.842	0.009
CRRT	114 (54.03%)	146 (49.16%)	χ^2^ = 1.171	0.279
Platelet transfusion	47 (22.27%)	30 (10.10%)	χ^2^ = 14.216	<0.001
Red blood cell transfusion	95 (45.02%)	115 (38.72%)	χ^2^ = 2.021	0.155
Plasma infusion	118 (55.92%)	111 (37.37%)	χ^2^ = 17.146	<0.001
Infusion of cold precipitated clotting factors	24 (11.37%)	18 (6.06%)	χ^2^ = 4.593	0.032
Infusion of human albumin	157 (74.41%)	209 (70.37%)	χ^2^ = 0.998	0.318
Hospitalization status				
ICU Stay (Days)	7.00 (3.00,14.00)	5.00 (3.00,13.00)	Z = 1.661	0.095
Length of hospital stay (Days)	16.00 (8.00,29.00)	16.00 (9.00,28.00)	Z = 0.185	0.854

Z, Mann–Whitney test, χ^2^, Chi-square test.

M, Median; Q₁, 1st Quartile; Q₃, 3st Quartile.

SALI, sepsis-associated liver injury; APACHE II, acute physiology and chronic health evaluation II; SOFA, sequential organ failure assessment; HR, heart rate; SBP, systolic blood pressure; DBP, diastolic blood pressure; MAP, mean arterial pressure; RR, respiratory rate; AB, actual bicarbonate; OI, oxygenation index; TP, total protein; ALB, albumin; TBIL, total bilirubin; DBIL, direct bilirubin; ALT, alanine aminotransferase; AST, aspartate aminotransferase; K, potassium; Na, sodium; Cl, chloride; Ca, calcium; PO_4_, phosphorus; Mg, magnesium; BUN, blood urea nitrogen; Cr, creatinine; PCT, procalcitonin; PT, prothrombin time; PT-INR, prothrombin time-international normalization ratio; APTT, activated partial thromboplastin time; FIB, fibrinogen; TT, thrombin time, WBC, white blood cells; NEU, neutrophils; LYM, lymphocytes; MON, monocytes; Hb, hemoglobin; RDW, red blood cell distribution width; PLT, platelet; PDW, platelet distribution width; P-LCR, platelet large cell ratio; MPV, mean platelet volume; CRRT, continuous renal replacement therapy; ICU, intensive care unit.

### 3.2 Feature selection

**Figure 2:**
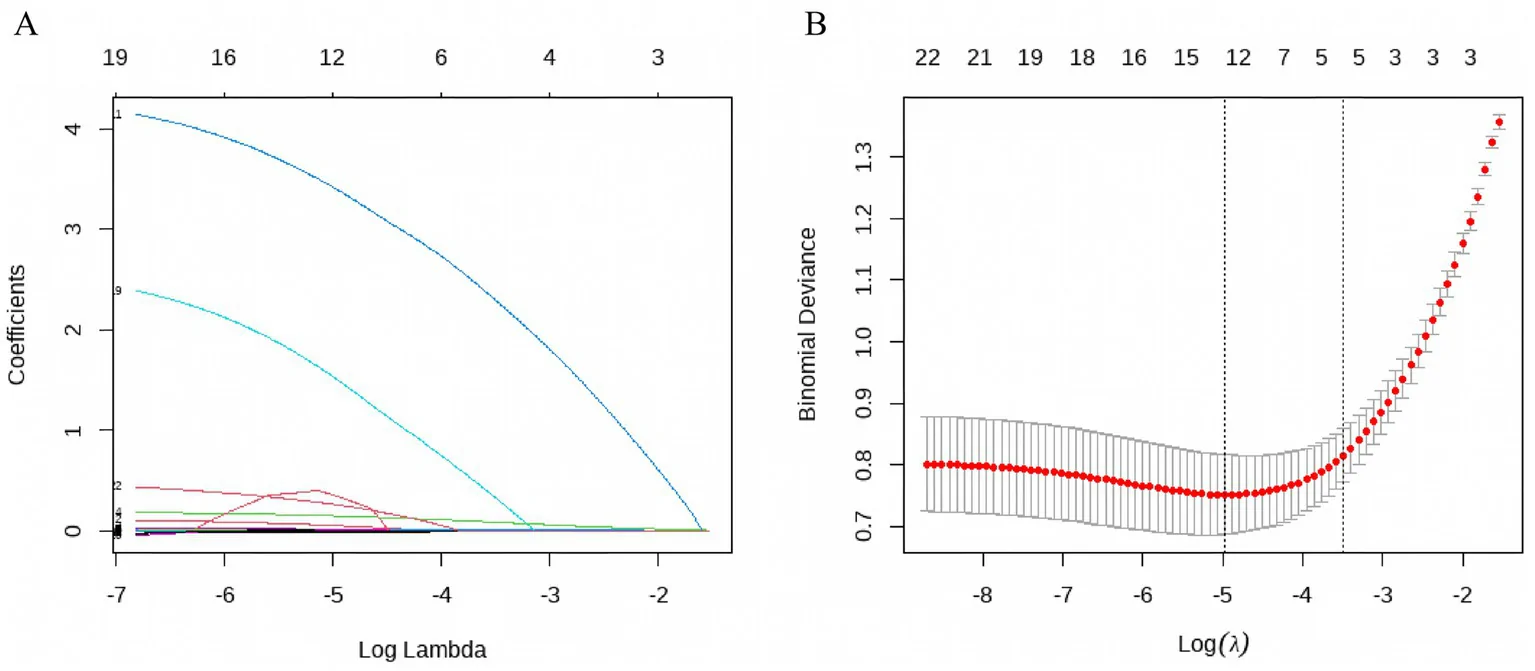
LASSO regression–based variable selection. **(A)** Coefficient profiles of candidate predictors. **(B)** Ten-fold cross-validation curve for selection of the optimal regularization parameter *λ*. LASSO, Least absolute shrinkage and selection operator.

Of the 71 candidate predictors evaluated in the training cohort, 24 met the exploratory univariable screening criterion of *p* < 0.05 and were entered into LASSO regression. LASSO regression was subsequently performed for coefficient shrinkage and further variable selection. Ten-fold cross-validation was used to select the penalty parameter corresponding to the minimum cross-validated error (λ_min = 0.006894539). Figure 2A shows the coefficient profiles of the candidate predictors during LASSO regularization, and Figure 2B shows the ten-fold cross-validation procedure used to select λmin. At λmin = 0.006894539, 12 candidate variables had nonzero coefficients: age, body temperature, DBIL, AST, serum sodium, blood urea nitrogen, creatinine, PT-INR, platelet count, plateletcrit, vasoactive-drug use, and cryoprecipitate transfusion.

The 12 variables retained by LASSO were entered into forward stepwise multivariable logistic regression, using entry and removal criteria of *α* = 0.05 and α = 0.10, respectively, to derive the final prediction model. The results indicated that age (OR, 0.975; 95% CI, 0.958–0.992; *p* = 0.005), DBIL (OR, 1.196; 95% CI, 1.143–1.250; *p* < 0.001), AST (OR, 1.010; 95% CI, 1.006–1.014; *p* < 0.001), PT-INR (OR, 57.507; 95% CI, 18.583–177.965; *p* < 0.001), and vasoactive-drug use (OR, 8.685; 95% CI, 1.015–74.320; *p* = 0.048) were retained as predictors associated with subsequent in-hospital SALI. The estimate for vasoactive-drug use was imprecise, as indicated by its extremely wide confidence interval and borderline statistical significance. Detailed model parameters are presented in Table 2.

**Table 2:** Final multivariable logistic regression model developed in the training cohort for predicting subsequent in-hospital SALI.

Predictor	Coding or unit	β	SE	Wald χ^2^	*P*	OR (95% CI)
Age	Per 1-year increase	−0.025	0.009	7.997	0.005	0.975 (0.958–0.992)
DBIL	Per 1 μmol/L increase	0.179	0.023	61.091	<0.001	1.196 (1.143–1.250)
AST	Per 1 U/L increase	0.010	0.002	25.915	<0.001	1.010 (1.006–1.014)
PT-INR	Per 1.0-unit increase	4.052	0.576	49.421	<0.001	57.507 (18.583–177.965)
Vasoactive-drug use	0 = no; 1 = yes	2.162	1.095	3.895	0.048	8.685 (1.015–74.320)
Intercept	—	−9.119	1.565	33.969	<0.001	—

β, regression coefficient; SE, standard error; OR, odds ratio; CI, confidence interval; SALI, sepsis-associated liver injury; DBIL, direct bilirubin; AST, aspartate aminotransferase; PT-INR, prothrombin time–international normalized ratio. Vasoactive-drug use was coded as 0 for no and 1 for yes. ORs for continuous predictors represent the change in odds associated with a one-unit increase in the corresponding predictor.

### 3.3 Model diagnostics

Diagnostic analyses were performed in the training cohort. The median PT-INR was 1.35 (IQR, 1.21–1.52; range, 0.90–3.36). Restricted cubic spline analyses showed no statistically significant evidence of nonlinearity for age (*p* for nonlinearity = 0.572), DBIL (*p* for nonlinearity = 0.498), AST (*p* for nonlinearity = 0.062), or PT-INR (*p* for nonlinearity = 0.172); these predictors were therefore retained as linear continuous terms on their original measurement scales. VIFs ranged from 1.01 to 1.22, indicating no important multicollinearity in the final model. Twenty-nine observations exceeded the Cook’s distance threshold of 4/*n*. After excluding these observations and refitting the model, the direction and magnitude of the regression coefficients remained materially unchanged.

### 3.4 Nomogram construction

**Figure 3:**
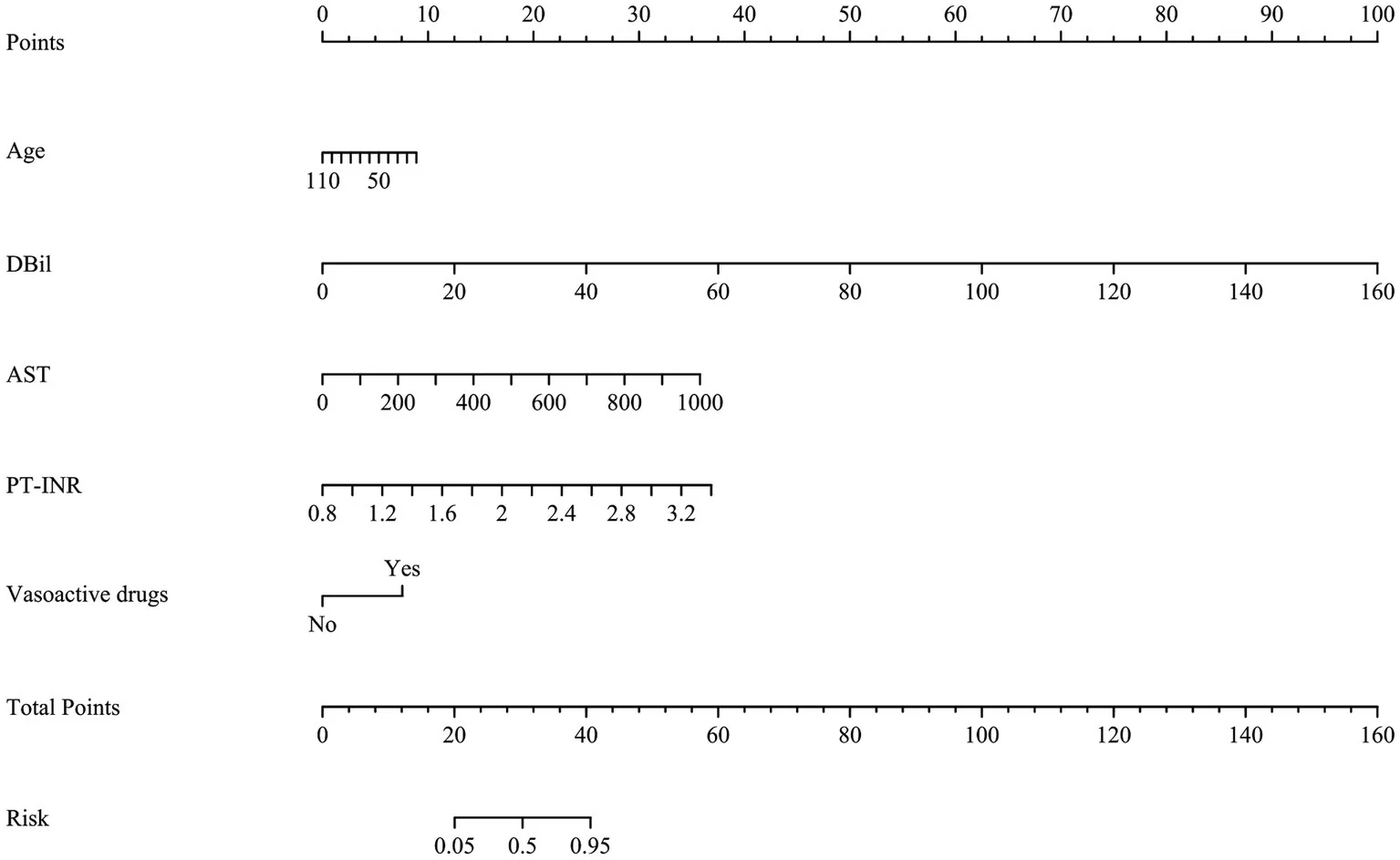
Nomogram for estimating the probability of subsequent in-hospital SALI in patients with sepsis. Age is expressed in years, DBIL in μmol/L, AST in U/L, and PT-INR as a unitless ratio; vasoactive-drug use is coded as no or yes. SALI, Sepsis-associated liver injury; DBil, Direct bilirubin; AST, Aspartate aminotransferase; PT-INR, Prothrombin time-international normalization ratio.

A nomogram was developed from the five predictors retained in the final multivariable logistic regression model (Figure 3). For an individual patient, each predictor value corresponds to a point score on the nomogram. The predictor-specific scores are summed and mapped to the probability scale to estimate the risk of subsequent in-hospital SALI.

By translating the multivariable logistic regression equation into a graphical format, the nomogram provides a convenient method for estimating an individual patient’s probability of subsequent in-hospital SALI. It displays the relative statistical weight of each predictor within the prediction equation, but these weights should not be interpreted as causal contributions to the occurrence of SALI. The nomogram is intended to support risk estimation and early risk stratification. The present study did not evaluate whether use of the nomogram changes clinical decisions, guides effective interventions, or improves patient outcomes.

The complete prediction equation was derived from the final multivariable logistic regression model. The outcome was coded as 1 for subsequent in-hospital SALI and 0 for no SALI during hospitalization. For an individual patient, the linear predictor was calculated as: LP = −9.119 − 0.025 × Age + 0.179 × DBIL + 0.010 × AST + 4.052 × PT-INR + 2.162 × Vasoactive-drug use, where age is expressed in years, DBIL in μmol/L, AST in U/L, PT-INR as a unitless ratio, and vasoactive-drug use is coded as 1 if at least one prespecified vasoactive agent was administered within the predictor-assessment window and 0 otherwise. The predicted probability of subsequent in-hospital SALI was calculated as P(SALI) = 1/[1 + exp.(−LP)].

### 3.5 Model performance validation

#### 3.5.1 External geographic validation cohort

At Chongqing General Hospital, 520 patients with sepsis were screened during the study period. Of these, 96 were excluded: 5 were younger than 18 years, 33 died within 24 h after ICU admission or had an ICU stay shorter than 24 h, 42 had chronic liver disease or baseline hepatic dysfunction, 3 had confirmed drug-induced liver injury, and 13 had missing required data. The external geographic validation cohort therefore included 424 patients, of whom 183 developed subsequent in-hospital SALI (43.2%) (Figure 1). The distributions of the final-model predictors according to SALI status are presented in Supplementary Table S2.

#### 3.5.2 Discrimination

**Figure 4:**
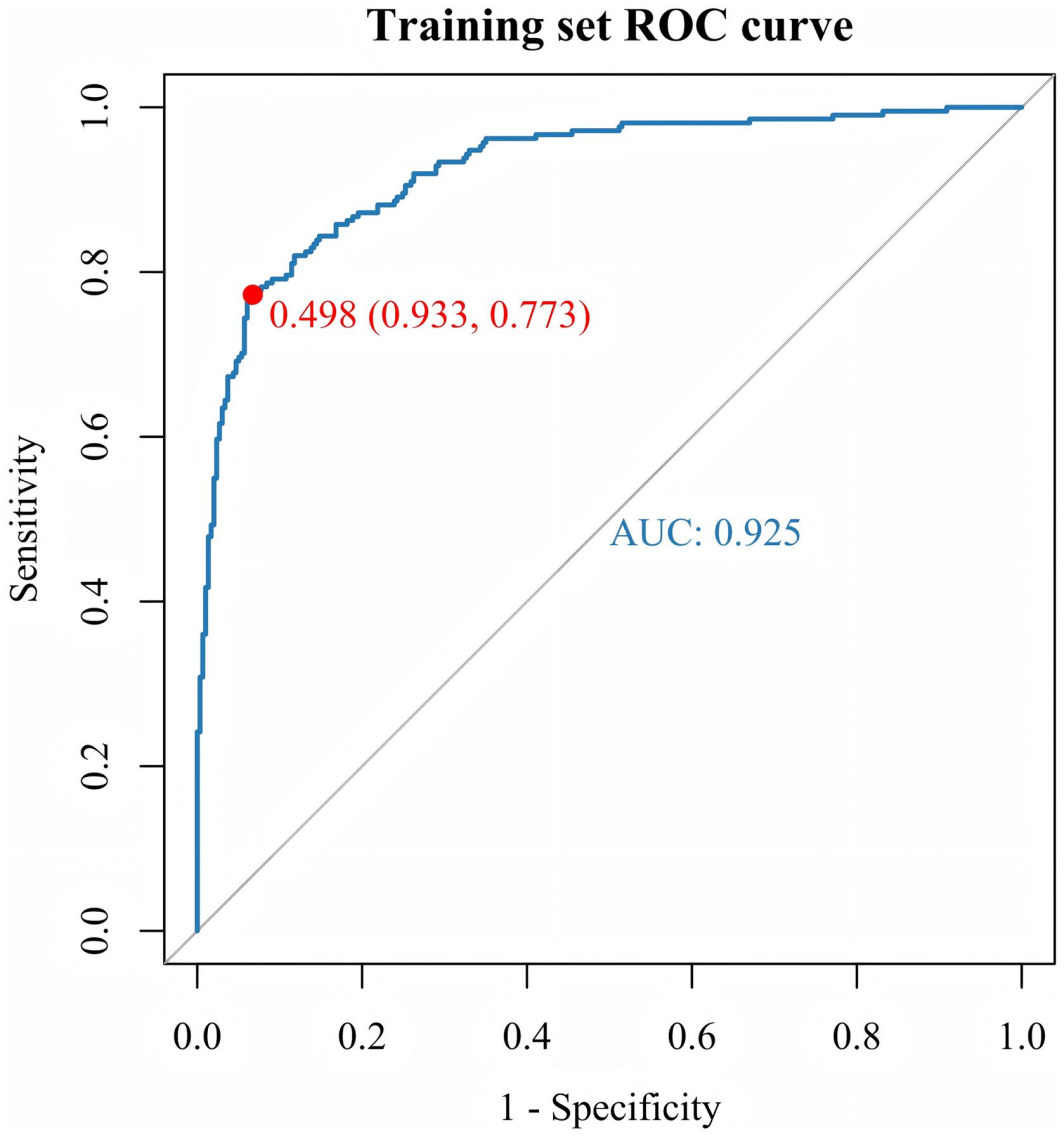
ROC curve of the nomogram model in the training cohort. The area under the curve was 0.925 (95% CI, 0.901–0.948). The highlighted point represents the training-derived cutoff of 0.498, with specificity of 0.933 and sensitivity of 0.773. ROC, receiver operating characteristic; AUC, area under the curve; CI, confidence interval.

The nomogram achieved AUCs of 0.925 (95% CI, 0.901–0.948), 0.918 (95% CI, 0.888–0.948), and 0.884 (95% CI, 0.849–0.918) in the training, testing, and external geographic validation cohorts, respectively, indicating favorable discrimination across the three cohorts. The corresponding ROC curves are presented in Figure 4 and Supplementary Figures S1A,B. Cohort-specific discrimination and fixed-cutoff classification metrics are summarized in Table 3.

**Table 3:** Discrimination and classification performance of the nomogram across the training, testing, and external geographic validation cohorts using the fixed training-derived cutoff.

Dataset	AUC (95% CI)	Fixed probability cutoff	Sensitivity (95% CI)	Specificity (95% CI)	PPV (95% CI)	NPV (95% CI)
Training cohort (*n* = 508; SALI/non-SALI = 211/297)	0.925 (0.901–0.948)	0.498	0.773 (0.711–0.824)	0.933 (0.898–0.956)	0.891 (0.837–0.928)	0.852 (0.810–0.887)
Testing cohort (*n* = 339; SALI/non-SALI = 141/198)	0.918 (0.888–0.948)	0.498	0.752 (0.674–0.816)	0.909 (0.861–0.942)	0.855 (0.782–0.906)	0.837 (0.782–0.881)
External geographic validation cohort (*n* = 424; SALI/non-SALI = 183/241)	0.884 (0.849–0.918)	0.498	0.634 (0.562–0.700)	0.950 (0.915–0.971)	0.906 (0.843–0.946)	0.774 (0.723–0.818)

AUC, area under the receiver operating characteristic curve; CI, confidence interval; PPV, positive predictive value; NPV, negative predictive value. The probability cutoff of 0.498 was selected exclusively in the training cohort by maximizing the Youden index and was applied unchanged to the testing and external geographic validation cohorts. Sensitivity, specificity, PPV, and NPV were calculated at this fixed cutoff, with 95% CIs estimated using the Wilson method. Cohort labels report the total sample size and the corresponding numbers of patients with and without subsequent in-hospital SALI. The probability cutoff of 0.498 was selected exclusively in the training cohort by maximizing the Youden index and was applied unchanged to the testing and external geographic validation cohorts. Sensitivity, specificity, PPV, and NPV were calculated at this fixed cutoff, with 95% CIs estimated using the Wilson score method. The cutoff represents a data-derived operating threshold for performance description and should not be interpreted as a universally validated clinical intervention threshold. The cutoff was used as a data-derived operating threshold for performance description and should not be interpreted as a universally validated clinical intervention threshold.

#### 3.5.3 Calibration performance

**Figure 5:**
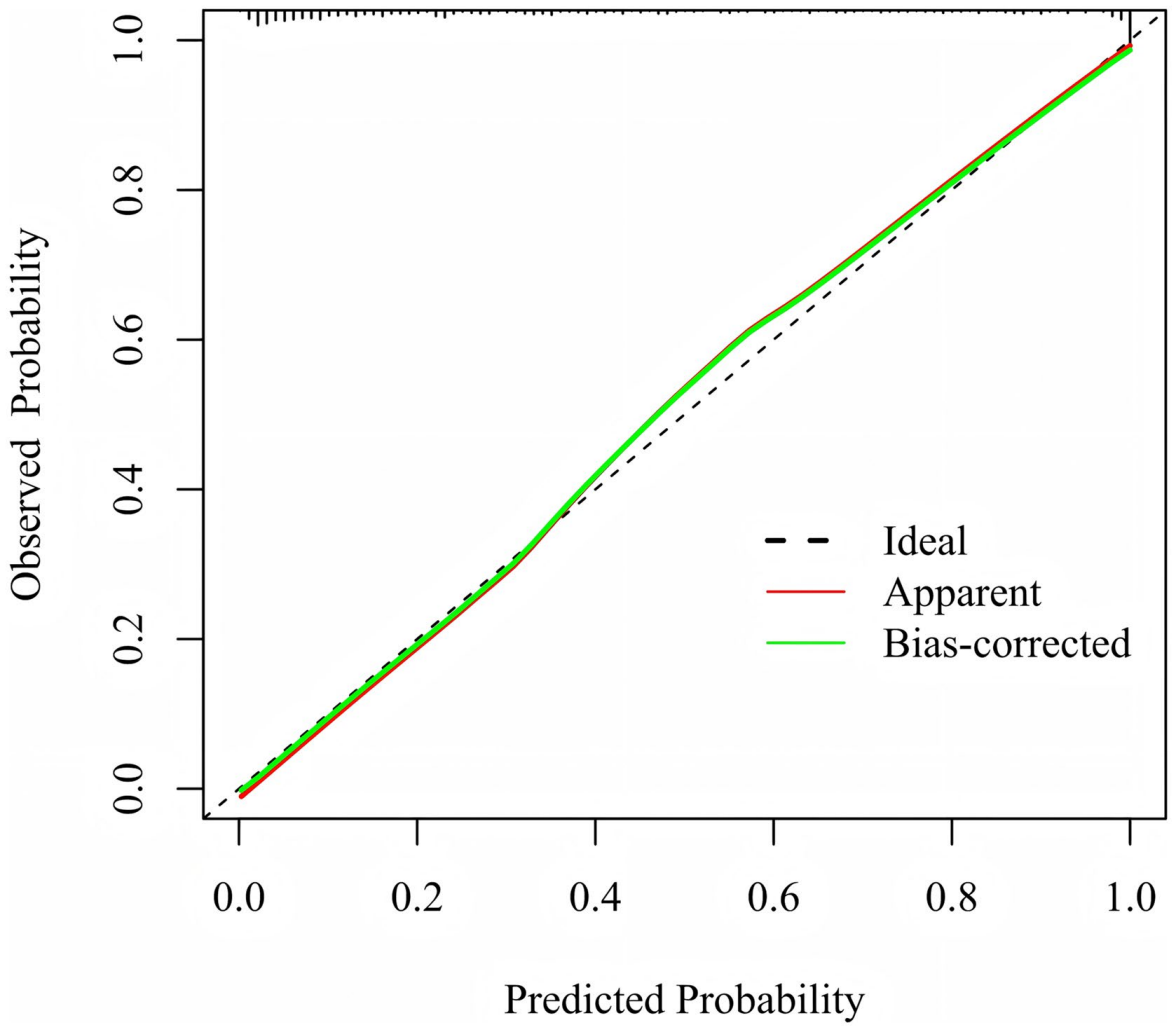
Calibration curves of the nomogram model in the training cohort. The x-axis represents predicted probability and the y-axis represents observed probability. The dashed line indicates perfect calibration, the apparent curve represents the original model performance, and the bias-corrected curve was obtained using 1,000 bootstrap resamples. Calibration-in-the-large was 0.024 and the calibration slope was 0.953.

Calibration performance differed across cohorts. In the training cohort, the calibration-in-the-large was 0.024 and the calibration slope was 0.953, indicating calibration close to the ideal values. In the testing cohort, the corresponding values were 0.025 and 0.643. In the external geographic validation cohort, the calibration-in-the-large was 0.945 and the calibration slope was 0.600, indicating substantial calibration drift in the external population. The Brier scores were 0.105 (95% CI, 0.090–0.124), 0.110 (95% CI, 0.089–0.132), and 0.140 (95% CI, 0.115–0.166) in the training, testing, and external geographic validation cohorts, respectively. Cohort-specific calibration plots are shown in Figure 5 and Supplementary Figures S2A,B, and the complete calibration metrics are summarized in Table 4.

**Table 4:** Calibration performance of the nomogram across the training, testing, and external geographic validation cohorts.

Dataset	Brier score (95% CI)	Calibration-in-the-large	Calibration slope
Training cohort (*n* = 508)	0.105 (0.090–0.124)	0.024	0.953
Testing cohort (*n* = 339)	0.110 (0.089–0.132)	0.025	0.643
External geographic validation cohort (*n* = 424)	0.140 (0.115–0.166)	0.945	0.600

CI, confidence interval. Ideal values are 0 for calibration-in-the-large and 1 for the calibration slope. The 95% CIs for the Brier scores were estimated using 2,000 nonparametric bootstrap resamples. Confidence intervals were not estimated for calibration-in-the-large or calibration slope.

#### 3.5.4 Decision curve analysis

**Figure 6:**
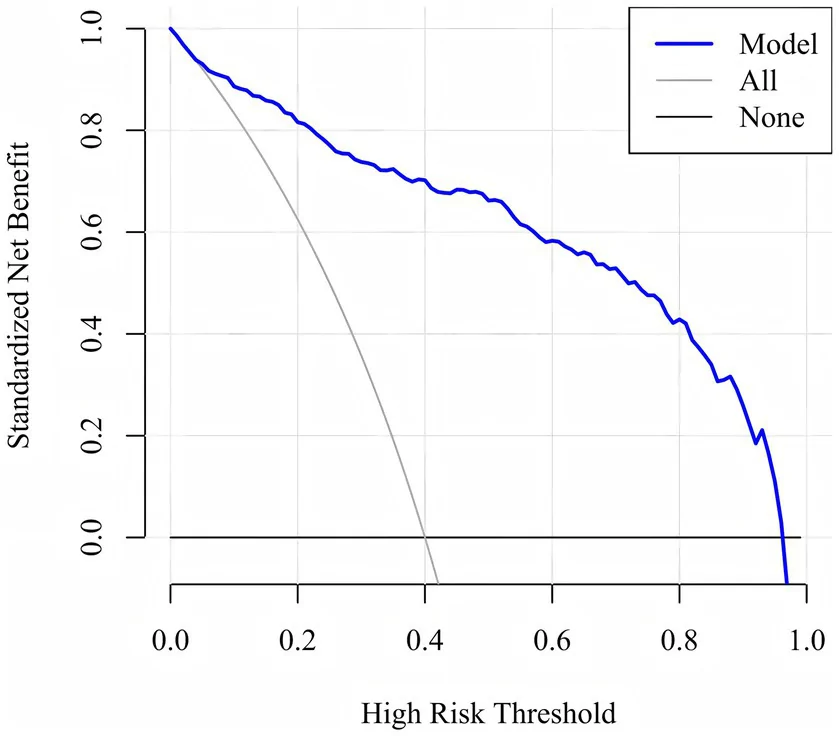
DCA curves of the nomogram model in the training cohort. The x-axis represents threshold probability, and the y-axis represents standardized net benefit. The model, “All,” and “None” curves represent model-guided action, action for all patients, and action for no patients, respectively. Although the full mathematical threshold range is displayed, clinical interpretation was restricted to the illustrative range of 0.10–0.40.

DCA was performed in the training, testing, and external geographic validation cohorts to evaluate the potential decision-analytic value of the nomogram (Figure 6; Supplementary Figures S3A,B). The x-axis represents the threshold probability at which model-guided clinical action would be considered, whereas the y-axis represents the standardized net benefit. Although the model curve showed a positive standardized net benefit over a broad mathematical range of threshold probabilities, not all thresholds were considered equally meaningful in clinical practice. Accordingly, clinical interpretation was restricted to the more plausible threshold-probability range of 0.10–0.40. Within this interval, the model curves in all three cohorts remained above both the “All” and “None” reference strategies, indicating potential net benefit when the predicted risk is used to identify patients for intensified laboratory surveillance and clinical reassessment. Such actions may include closer monitoring of liver biochemical and coagulation indices, review of potentially hepatotoxic medications, and reassessment of infection control, hemodynamic status, and organ perfusion. The 0.10–0.40 interval should be regarded as an illustrative decision range rather than a prospectively validated or universally applicable clinical threshold.

#### 3.5.5 Classification performance at the fixed cutoff

The probability cutoff that maximized the Youden index in the training cohort was 0.498 and was applied unchanged to the testing and external geographic validation cohorts. At this fixed cutoff, the sensitivity, specificity, PPV, and NPV were 0.773, 0.933, 0.891, and 0.852 in the training cohort; 0.752, 0.909, 0.855, and 0.837 in the testing cohort; and 0.634, 0.950, 0.906, and 0.774 in the external geographic validation cohort, respectively. The corresponding 95% confidence intervals are presented in Table 3.

#### 3.5.6 Comparison with the SOFA-only benchmark model

The nomogram demonstrated higher discrimination than the SOFA-only benchmark model. In the training cohort, the AUCs were 0.925 for the nomogram and 0.544 for the SOFA-only model (ΔAUC = 0.381; DeLong *p* < 0.001). In the testing cohort, the corresponding AUCs were 0.918 and 0.553 (ΔAUC = 0.365; DeLong *p* < 0.001). The integrated discrimination improvement values were 0.553 and 0.576 in the training and testing cohorts, respectively.

## 4 Discussion

Using multicenter real-world clinical data, we developed and externally validated a nomogram for estimating the risk of subsequent in-hospital SALI in patients with sepsis. The model demonstrated favorable discrimination across the training, testing, and external geographic validation cohorts. However, calibration was less stable outside the training cohort: the calibration slope decreased from 0.953 in the training cohort to 0.643 in the testing cohort and 0.600 in the external geographic validation cohort, while the external calibration-in-the-large of 0.945 indicated substantial calibration drift. These findings suggest that local recalibration may be required before the model is applied in other clinical settings. Because the recruitment periods overlapped, the third-center validation primarily assessed geographic transportability across institutions and patient case mixes rather than temporal transportability to a strictly later population. Decision curve analysis further suggested potential decision-analytic value; however, the broad mathematical range over which the model showed a positive standardized net benefit should not be interpreted as uniformly clinically meaningful. Based on the intended use of the model, clinical interpretation was restricted to threshold probabilities of 0.10–0.40. Within this range, a high-risk classification could reasonably prompt intensified monitoring of liver biochemical and coagulation indices, medication review, and reassessment of infection control, hemodynamic status, and organ perfusion rather than mandate a specific SALI-directed treatment. Because this threshold range has not been evaluated in a prospective impact study, it should be considered illustrative and should not be interpreted as a universally validated clinical decision threshold.

SALI is a heterogeneous manifestation of sepsis-associated organ dysfunction that may involve dysregulated inflammation, impaired hepatic microcirculation, cholestasis, coagulation abnormalities, and altered cellular metabolism (15–17). These overlapping processes provide biological context for the predictors retained in the model, but the present study was designed to estimate risk rather than to establish the mechanisms or causal pathways leading to SALI.

Routine liver biochemical tests and general organ-dysfunction scores provide important information regarding established hepatic abnormalities and overall sepsis severity, but they are not individualized prediction tools for subsequent SALI. The present nomogram was therefore developed to integrate several routinely available variables into a single patient-level risk estimate rather than to replace biochemical testing, clinical assessment, or established severity scores.

The five variables retained in the final model represent demographic, hepatobiliary, hepatocellular-injury, coagulation, and circulatory-support domains. The inverse association between age and subsequent in-hospital SALI was unexpected and should be interpreted cautiously. It may reflect differences in sepsis phenotype, comorbidity burden, case mix, disease severity, or clinical testing patterns rather than a direct protective effect of increasing age. Higher DBIL, AST, and PT-INR values may summarize evolving hepatobiliary dysfunction, hepatocellular injury, impaired hepatic synthetic function, and systemic coagulation abnormalities (18–20). These variables should therefore be regarded as predictive markers rather than causal determinants. In particular, because DBIL contributes to TBIL and AST reflects the same hepatocellular-injury domain as ALT, their biological proximity to the outcome definition may have contributed to optimistic estimates of model performance, despite being measured before SALI onset (18, 19).

Vasoactive drugs are commonly administered to patients with septic shock to support arterial pressure and tissue perfusion when clinically indicated (21, 22). However, vasoactive-drug use requires particular caution in the present model because its estimated association was borderline and highly imprecise (OR = 8.685, 95% CI, 1.015–74.320; *p* = 0.048). Rather than indicating that vasoactive therapy directly causes hepatic injury, this variable may primarily reflect shock severity, hemodynamic instability, impaired tissue perfusion, and treatment intensity. Residual confounding related to drug type, dose, duration, fluid resuscitation, treatment response, and shock phenotype cannot be excluded. Accordingly, the observed association should not be interpreted as a reason to delay or withhold clinically indicated vasoactive therapy. Instead, the need for vasoactive support may identify patients who warrant closer assessment of hepatic function, coagulation status, and systemic perfusion.

The SALI outcome definition used in this study was prespecified and adopted from a previously published SALI prediction study rather than derived from the present data. It should be regarded as an operational composite biochemical outcome rather than a universally accepted diagnostic standard for SALI. Neither TBIL nor ALT, which directly defined the outcome, was included in the final model. Nevertheless, DBIL is a component of TBIL, and AST and ALT are both biomarkers of hepatocellular injury. Although DBIL and AST were measured before the first occurrence of SALI, elevated values may already reflect evolving subclinical hepatic dysfunction before the prespecified operational outcome thresholds are reached. These variables should therefore be interpreted as temporally antecedent but biologically proximal risk markers, rather than as independent causal determinants of SALI. Accordingly, the nomogram should not be interpreted as demonstrating incremental predictive value beyond the existing TBIL- and ALT-based outcome criteria.

Two recently published models provide direct context for the findings of the present study. Tao et al. developed a ferritin-centered nomogram using 1,109 patients with sepsis from the MIMIC-IV database and clinically validated it in 122 patients from a single hospital. Their model included mechanical ventilation, continuous renal replacement therapy, vasoactive-agent use, alkaline phosphatase, INR, and serum ferritin and achieved AUCs of 0.765 and 0.773 in the development and validation cohorts, respectively (12). Their modeling strategy combined LASSO and Boruta feature selection, followed by multivariable logistic regression and nomogram construction. Importantly, their study applied the same operational composite biochemical definition as the present study—TBIL >34.2 μmol/L and/or ALT >80 U/L—thereby facilitating conceptual comparison of the predicted outcomes. Their study highlighted a ferritin-centered biological perspective integrating inflammatory and iron-metabolism information.

Lei et al. adopted a substantially different strategy using 8,834 patients from MIMIC-IV for model development and internal validation and 4,236 patients from eICU-CRD for external validation (23). They compared nine machine-learning algorithms and developed a stacking ensemble model interpreted using SHAP values. The stacking model achieved AUCs of approximately 0.995, 0.838, and 0.721 in the training, internal validation, and external validation cohorts, respectively. Total bilirubin, lactate, prothrombin time, alanine aminotransferase, mechanical ventilation, and vasoactive-drug use were among the influential features. However, their SALI definition—INR > 1.5 combined with total bilirubin >2 mg/dL within one week of ICU admission—differed substantially from the biochemical definition used in our study. Their analysis also incorporated 42 predictors and complex ensemble algorithms, providing greater modeling flexibility at the cost of increased implementation complexity.

The present study provides a complementary approach by developing a parsimonious five-predictor logistic-regression nomogram using routinely available variables from three hospital electronic medical-record systems and evaluating it in an independent hospital-based geographic validation cohort. The nomogram achieved AUCs of 0.925, 0.918, and 0.884 in the training, testing, and external geographic validation cohorts, respectively. However, these values should not be interpreted as direct superiority because the studies differed in outcome definition, event incidence, predictor timing, case mix, candidate-variable sets, and validation design. Across studies, hepatic biochemical abnormalities, coagulation dysfunction, and circulatory-support requirements nevertheless emerged as recurring predictive domains.

This study has several strengths. It included 1,271 patients from three hospitals, used separate training and testing cohorts, and reserved a third hospital for external geographic validation. The final nomogram contained only five routinely available variables and was presented with its complete regression equation, facilitating independent calculation and future validation. The structured comparison with existing models further shows that the present approach prioritizes parsimony and routine clinical availability rather than algorithmic complexity.

Several limitations should be considered. First, the retrospective complete-case design may have introduced selection and information bias. Variable-specific missing counts and missingness patterns were not retained, and patients with incomplete required data were excluded. Although all predictors preceded formal SALI onset, the exact interval from T0 to SALI was not retained, preventing evaluation at fixed prediction horizons. DBIL and AST may also reflect evolving subclinical hepatic dysfunction because of their biological proximity to the outcome definition. This predictor–outcome proximity may have contributed to optimistic discrimination. A *post hoc* model excluding DBIL was not developed; therefore, the extent to which the reported performance depended on DBIL could not be quantified. Fixed-horizon landmark analyses and sensitivity analyses excluding patients with early elevations of liver-related biomarkers were also not performed. Because no universally accepted SALI definition is currently available, the present findings should be interpreted as specific to the prespecified composite biochemical outcome used in this study. Sensitivity analyses using bilirubin-only criteria, a SOFA liver subscore–based definition, or other liver-dysfunction or liver-failure definitions were not performed. Therefore, the discrimination, calibration, and potential clinical utility of the model under alternative outcome definitions remain uncertain. The retained analytical dataset did not contain the complete patient-level temporal information required to reconstruct alternative outcome status and onset consistently across the three centers; therefore, predictor ascertainment could not be re-delimited under an alternative outcome framework during revision. Second, residual confounding related to illness severity, shock phenotype, treatment strategies, and ICU management practices cannot be excluded. The associations identified in this prediction study should not be interpreted causally. In particular, the estimate for vasoactive-drug use was borderline and highly imprecise and may primarily reflect shock severity and treatment intensity. Third, the model was based on static early measurements, and dynamic changes and predictor interactions were not evaluated. The sequential use of univariable screening, LASSO selection, and stepwise regression may have introduced model-selection instability and optimistic performance estimates. Although the conventional EPV was 42.2 for the five-predictor final model, it does not capture the additional complexity introduced by the preceding selection procedures. Finally, patients from the two development hospitals were pooled, and center-specific SALI incidence, baseline characteristics, and outcome timing were not retained separately. Consequently, formal adjustment for development-center effects and leave-one-center-out validation could not be performed using the retained pooled analytical dataset. Although common eligibility criteria, variable definitions, coding procedures, predictor-assessment rules, and fixed SALI thresholds were applied across centers, laboratory platforms, local reference intervals, and routine ICU treatment protocols were not formally harmonized. Differences in case mix, laboratory measurement procedures, and local clinical practice may therefore have contributed to between-center heterogeneity and the calibration drift observed in the external geographic validation cohort. Because the recruitment periods overlapped, the third-center cohort provided geographic rather than strict temporal validation. Further prospective geographic and temporal validation is required before clinical implementation.

## 5 Conclusion

This study developed and externally validated a nomogram for estimating the risk of subsequent in-hospital SALI in patients with sepsis. The model demonstrated favorable discrimination across the training, testing, and external geographic validation cohorts, although calibration deteriorated outside the training cohort. The nomogram may provide a practical approach to risk estimation and early risk stratification, particularly for identifying patients who may warrant closer monitoring and clinical reassessment. Because this was a retrospective observational prediction study, the associations of the included predictors should not be interpreted as causal. The present findings do not establish that implementation of the nomogram improves prognosis, guides effective interventions, or optimizes ICU resource allocation. Local recalibration, prospective validation, and clinical-impact evaluation are required before routine clinical use.

## Data Availability

The data that support the findings of this study are available from the corresponding author upon reasonable request. Requests to access these datasets should be directed to RT, 1031829196@qq.com.
