# Supplementary material for: Development and validation of a nomogram for early prediction of sepsis-associated liver injury: a multicenter study
Source: Front Med (Lausanne). 2026 Aug 21;13:1899424. doi: 10.3389/fmed.2026.1899424 (PMC13542648; doi:10.3389/fmed.2026.1899424)
Supplement: Supplementary file 1 [file Data_Sheet_1.pdf]

TRIPOD Checklist: Prediction Model Development and Validation

Manuscript: Development and Validation of a Nomogram for Early Prediction of Sepsis-Associated Liver Injury: A Multicenter Study

Completion note. Based on the TRIPOD 2015 development-and-validation checklist. Stable section, table, and figure references are used because pagination may change. D = development; V = validation. Status categories: Reported; Reported with data limitation disclosed; Partially reported; Not explicitly reported; Not applicable.

Section / topic	Item	TRIPOD reporting requirement	Status	Location in revised manuscript
**Title and abstract**	**1 (D;V)**	Identify the report as a study developing and validating a multivariable prediction model, and state the target population and predicted outcome.	Reported	Title page: the title identifies development and validation of a nomogram for SALI in patients with sepsis.
**Title and abstract**	**2 (D;V)**	Summarize objectives, design, setting, participants, sample size, predictors, outcome, statistical methods, results, and conclusions.	Reported	Abstract: Background, Methods, Results, and Conclusions.
**Introduction**	**3a (D;V)**	Explain the clinical context, whether the model is diagnostic or prognostic, the rationale for the study, and relevant existing prediction models.	Reported	Introduction, paragraphs 1-4; Discussion, structured comparison with Tao et al. and Lei et al.
**Introduction**	**3b (D;V)**	State the objectives and clarify that the study includes both model development and validation.	Reported	Introduction, final paragraph.
**Methods - Source of data**	**4a (D;V)**	Describe the study design and data source separately for development and validation cohorts.	Reported	Methods 2.1, Data Source.
**Methods - Source of data**	**4b (D;V)**	Report key study dates, including recruitment start/end and the end of outcome ascertainment or follow-up.	Reported	Methods 2.1-2.2: recruitment periods for all centers; outcome ascertainment from T0 to first SALI event or hospital discharge.
**Methods - Participants**	**5a (D;V)**	Describe the study setting, level of care, number of centers, and center locations.	Reported	Methods 2.1-2.2: three hospital ICUs in Hebei and Chongqing, China.
**Methods - Participants**	**5b (D;V)**	Describe participant eligibility criteria.	Reported	Methods 2.2, Study Population; Figure 1.
**Methods - Participants**	**5c (D;V)**	Describe treatments received when relevant to the prediction study.	Reported	Methods 2.3 lists relevant clinical interventions and defines temporally eligible vasoactive-drug use; Limitations notes that routine center protocols were not formally standardized.
**Methods - Outcome**	**6a (D;V)**	Clearly define the predicted outcome, including how and when it was assessed.	Reported with data limitation disclosed	Methods 2.2-2.3; Supplementary Figure S4. Subsequent in-hospital SALI was assessed after T0 until the first qualifying result or discharge; exact elapsed T0-to-SALI intervals were not retained.
**Methods - Outcome**	**6b (D;V)**	Report whether outcome assessment was blinded to predictor information.	Not explicitly reported	Methods 2.2 describes retrospective ascertainment using objective laboratory thresholds, but blinding to predictor information is not explicitly stated.
**Methods - Predictors**	**7a (D;V)**	Define all predictors, including how and when they were measured.	Reported	Methods 2.3 and Table 1 list candidate predictors and general ascertainment rules; Supplementary Table S3 and Figure S4 give detailed definitions, coding, units, and timing for the final predictors.
**Methods - Predictors**	**7b (D;V)**	Report whether predictor assessment was blinded to outcome and to other predictors.	Not explicitly reported	Methods 2.3 describes retrospective extraction using prespecified timing rules, but blinding of predictor assessment is not explicitly stated.
**Methods - Sample size**	**8 (D;V)**	Explain how the study size was determined.	Not explicitly reported	Recruitment periods, eligibility criteria, complete-case requirements, and final sample counts are reported, but the manuscript does not explicitly state whether an a priori sample-size calculation was performed or how study size was determined.
**Methods - Missing data**	**9 (D;V)**	Describe how missing data were handled and provide details of any imputation.	Reported with data limitation disclosed	Methods 2.4 and Results 3.1: complete-case analysis; no imputation; numbers excluded for missing required data are reported by center. Variable-specific missing counts and missingness patterns were not retained.
**Methods - Statistical analysis**	**10a (D)**	Describe how predictors were handled in the analyses.	Reported	Methods 2.5; Supplementary Table S3. Continuous predictors were analyzed on original scales and categorical predictors were binary coded.
**Methods - Statistical analysis**	**10b (D)**	Specify the model type, all model-building procedures, predictor selection, and internal validation.	Reported	Methods 2.5; Results 3.2; Figure 2. Univariable screening, LASSO with ten-fold cross-validation, stepwise multivariable logistic regression, and bootstrap-corrected calibration.
**Methods - Statistical analysis**	**10c (V)**	For validation, describe how predictions were calculated.	Partially reported	Results 3.3 provides the complete equation and Results 3.5 reports testing and external geographic validation performance. The manuscript does not explicitly state that the original development intercept and coefficients were applied without refitting.

**Methods - Statistical analysis**	**10d (D;V)**	Specify all performance measures used and any model comparisons.	Reported	Methods 2.5; Results 3.5; Tables 3-4; Figures 4-6; Supplementary Figures S1-S3 and Table S4. AUC, fixed-cutoff metrics, calibration plots/statistics, Brier score, DCA, and SOFA benchmark comparison are reported.
**Methods - Statistical analysis**	**10e (V)**	Describe any model updating or recalibration performed during validation.	Not applicable	No model-updating or recalibration analysis was performed or reported; validation performance is presented for the fitted nomogram.
**Methods - Risk groups**	**11 (D;V)**	Describe how risk groups were created, if applicable.	Not applicable	No categorical risk groups were created; the nomogram provides continuous predicted probabilities.
**Methods - Development vs validation**	**12 (V)**	Identify differences between development and validation data in setting, eligibility, outcome, and predictors.	Reported	Methods 2.1-2.3; Results 3.1 and 3.5; Discussion and Limitations. The same eligibility, predictor, and outcome rules were used; the third center provided geographic rather than temporal validation; local platforms and routine protocols were not formally harmonized.
**Results - Participants**	**13a (D;V)**	Describe participant flow, numbers with and without the outcome, and follow-up time when available; a flow diagram may be used.	Reported with data limitation disclosed	Figure 1 and Results 3.1. Screening, exclusion, inclusion, and outcome counts are reported. The revised Figure 1 caption states that all included patients were free of SALI at T0. Exact T0-to-event distributions were unavailable and disclosed.
**Results - Participants**	**13b (D;V)**	Describe participant characteristics and the extent of missing predictor/outcome data.	Reported with data limitation disclosed	Table 1; Supplementary Tables S1-S2; Results 3.1; Methods 2.4. Characteristics and center-level missing-data exclusions are reported, but variable-specific missingness is unavailable.
**Results - Participants**	**13c (V)**	Compare important variable and outcome distributions between validation and development data.	Partially reported	Baseline characteristics are presented separately in Supplementary Tables S1-S2 and outcome incidence is reported by analytical cohort; no formal side-by-side statistical comparison of development versus external validation distributions is presented.
**Results - Model development**	**14a (D)**	Report the number of participants and outcome events used in each analysis.	Reported	Abstract Results; Results 3.1-3.2. Development/internal cohort n=847; training n=508 with 211 SALI events; testing n=339 with 141 events; external geographic validation n=424 with 183 events.
**Results - Model development**	**14b (D)**	Report unadjusted associations between candidate predictors and outcome, if performed.	Reported	Table 1 and Results 3.2: univariable comparisons and variables meeting the prespecified screening criterion.
**Results - Model specification**	**15a (D)**	Present the complete model, including all coefficients and the intercept, so individual predictions can be calculated.	Reported	Table 2; Results 3.4: complete coefficients, intercept, coding, units, linear predictor, and probability equation.
**Results - Model specification**	**15b (D)**	Explain how to use the model.	Reported	Figure 3 and Results 3.4; Supplementary Table S3 and corrected Supplementary Code S1.
**Results - Model performance**	**16 (D;V)**	Report model performance measures with confidence intervals where appropriate.	Reported	Results 3.5; Tables 3-4; Figures 4-6; Supplementary Figures S1-S3. AUCs, Brier scores, and fixed-cutoff classification metrics have confidence intervals where estimated; calibration-in-the-large and slopes are reported as point estimates.
**Results - Model updating**	**17 (V)**	Report results of model updating, if performed.	Not applicable	No model-updating analysis was performed.
**Discussion - Limitations**	**18 (D;V)**	Discuss study limitations, including representativeness, event numbers, missing data, and model-development limitations.	Reported	Discussion, Limitations: retrospective complete-case design, unavailable exact timing, predictor-outcome proximity, residual confounding, selection instability, center heterogeneity, geographic rather than temporal validation, and limited generalizability.
**Discussion - Interpretation**	**19a (V)**	Interpret validation performance in relation to development performance and other validation evidence.	Reported	Discussion: comparison of training, testing, and external geographic validation performance, including calibration drift; structured comparison with prior SALI models.
**Discussion - Interpretation**	**19b (D;V)**	Provide an overall interpretation considering objectives, limitations, similar studies, and other evidence.	Reported	Discussion: principal findings, predictive domains, comparison with Tao et al. and Lei et al., and explicit distinction between prediction and causation.
**Discussion - Implications**	**20 (D;V)**	Discuss potential clinical use and implications for future research.	Reported	Discussion, clinical implications; Conclusion. Model positioned for risk estimation and closer surveillance; local recalibration, prospective validation, and clinical-impact studies are required.
**Other information - Supplementary resources**	**21 (D;V)**	State availability of supplementary resources such as protocol, calculator, code, and data.	Reported	Supplementary Table S3, Supplementary Figure S4, corrected Supplementary Code S1, data dictionary, and Availability of data and materials statement.
**Other information - Funding**	**22 (D;V)**	Report funding sources and the role of funders.	Partially reported	Funding section reports the Chongqing medical scientific research project (2024MSXM091), but the role of the funder is not stated.
