# Supplementary material for: Development and validation of a nomogram for early prediction of sepsis-associated liver injury: a multicenter study
Source: Front Med (Lausanne). 2026 Aug 21;13:1899424. doi: 10.3389/fmed.2026.1899424 (PMC13542648; doi:10.3389/fmed.2026.1899424)
Supplement: Supplementary file 2 [file Data_Sheet_2.docx]

**Supplementary Code S1**

**R code for calculating individual predicted risk**

\# Individual risk calculator for subsequent in-hospital SALI

calculate_sali_risk <- function(

age,

dbil,

ast,

pt_inr,

vasoactive_drug

) {

if (any(!vasoactive_drug %in% c(0, 1))) {

stop("vasoactive_drug must be coded as 0 for no or 1 for yes.")

}

lp <- -9.119 -

0.025 * age +

0.179 * dbil +

0.010 * ast +

4.052 * pt_inr +

2.162 * vasoactive_drug

predicted_probability <- plogis(lp)

return(predicted_probability)

}

\# Example

example_risk <- calculate_sali_risk(

age = 65,

dbil = 10,

ast = 50,

pt_inr = 1.2,

vasoactive_drug = 1

)

print(example_risk)

**Supplementary Table**

Table S1. Comparison of baseline characteristics between the training and testing sets

| Variables | Training set (n=508) | Testing set (n=339) | Z/χ² | *P* |
| --- | --- | --- | --- | --- |
| Demographic data |  |  |  |  |
| Male, n(%) | 317(62.40%) | 208(61.36%) | χ2=0.094 | 0.759 |
| Age, SD | 74.00(63.00,82.00) | 73.00(62.00,81.00) | Z=0.601 | 0.548 |
| Underlying diseases, n(%) |  |  |  |  |
| Coronary atherosclerotic heart disease | 113(22.24%) | 75(22.12%) | χ2=0.002 | 0.967 |
| Hypertension | 221(43.50%) | 152(44.84%) | χ2=0.147 | 0.702 |
| Diabetes | 140(27.56%) | 97(28.61%) | χ2=0.112 | 0.738 |
| Chronic obstructive pulmonary disease | 70(13.78%) | 37(10.91%) | χ2=1.512 | 0.219 |
| Cerebrovascular disease | 188(37.01%) | 123(36.28%) | χ2=0.046 | 0.830 |
| Chronic kidney disease | 125(24.61%) | 89(26.25%) | χ2=0.292 | 0.589 |
| Malignancy | 68(13.39%) | 50(14.75%) | χ2=0.315 | 0.574 |
| History of surgery within 3 months | 245(48.23%) | 167(49.26%) | χ2=0.087 | 0.768 |
| Infection sites, n(%) |  |  |  |  |
| Pulmonary | 332(65.35%) | 244(71.98%) | χ2=4.098 | 0.043 |
| Abdominal | 199(39.17%) | 146(43.07%) | χ2=1.277 | 0.258 |
| Blood | 67(13.19%) | 43(12.68%) | χ2=0.046 | 0.831 |
| Urinary system | 95(18.70%) | 57(16.81%) | χ2=0.491 | 0.483 |
| Central nervous system | 6(1.18%) | 11(3.24%) | χ2=4.403 | 0.036 |
| Skin and soft tissue | 14(2.76%) | 4(1.18%) | χ2=2.428 | 0.119 |
| Other | 19(3.74%) | 13(3.83%) | χ2=0.005 | 0.944 |
| ICU disease severity scores, M (Q₁, Q₃) |  |  |  |  |
| APACHE score | 23.00(18.00,28.00) | 23.00(18.00,28.00) | Z=0.640 | 0.522 |
| SOFA score | 9.00(7.00,11.00) | 9.00(7.00,11.00) | Z=0.311 | 0.755 |
| Vital signs, M (Q₁, Q₃) |  |  |  |  |
| Temperature(℃) | 36.50(36.00,37.00) | 36.50(36.00,37.10) | Z=1.057 | 0.285 |
| HR(times/min) | 96.00(84.00,113.00) | 98.00(82.00,114.00) | Z=0.138 | 0.891 |
| SBP(mmHg) | 121.00(104.50,140.00) | 121.00(107.50,141.00) | Z=0.962 | 0.336 |
| DBP(mmHg) | 67.00(57.00,78.00) | 66.00(56.50,78.00) | Z=0.286 | 0.775 |
| MAP(mmHg) | 85.17(75.33,94.75) | 85.33(76.00,95.00) | Z=0.258 | 0.796 |
| RR(times/min) | 20.00(16.00,25.00) | 21.00(16.00,28.00) | Z=1.126 | 0.258 |
| Laboratory tests, M (Q₁, Q₃) |  |  |  |  |
| pH | 7.38(7.30,7.43) | 7.37(7.30,7.43) | Z=0.528 | 0.597 |
| lactate(mmol/L) | 2.30(1.51,4.20) | 2.20(1.60,3.80) | Z=0.852 | 0.394 |
| AB(mmol/L) | 21.10(17.20,24.83) | 20.40(17.10,24.85) | Z=1.014 | 0.311 |
| OI | 217.86(150.83,318.98) | 211.40(145.44,302.51) | Z=0.503 | 0.615 |
| TP(g/L) | 51.00(44.88,58.80) | 50.70(45.40,58.15) | Z=0.091 | 0.928 |
| ALB(g/L)(μmol/L) | 27.85(23.90,31.80) | 27.40(23.30,31.30) | Z=0.981 | 0.327 |
| TBIL(μmol/L) | 18.60(12.20,35.28) | 16.80(10.80,36.25) | Z=1.394 | 0.163 |
| DBIL(μmol/L) | 8.00(4.40,13.50) | 6.80(4.15,15.35) | Z=0.677 | 0.499 |
| ALT(U/L) | 26.00(14.00,63.00) | 30.40(15.00,85.50) | Z=2.087 | 0.037 |
| AST(U/L) | 42.00(24.87,96.78) | 49.00(26.90,115.00) | Z=1.363 | 0.173 |
| K(mmol/L) | 4.00(3.60,4.59) | 4.07(3.60,4.56) | Z=0.831 | 0.406 |
| Na(mmol/L) | 140.00(135.47,145.00) | 139.00(135.60,143.90) | Z=1.141 | 0.254 |
| Cl(mmol/L) | 105.00(101.00,110.03) | 105.00(100.75,109.85) | Z=0.601 | 0.548 |
| Ca(mmol/L) | 1.99(1.86,2.16) | 1.98(1.85,2.14) | Z=0.845 | 0.398 |
| PO_4_(mmol/L) | 1.08(0.82,1.39) | 1.17(0.79,1.48) | Z=1.506 | 0.132 |
| Mg(mmol/L) | 0.82(0.71,0.94) | 0.83(0.68,0.95) | Z=0.191 | 0.849 |
| BUN(mmol/L) | 12.71(8.00,20.15) | 11.80(7.62,20.04) | Z=0.976 | 0.329 |
| Cr(μmol/L) | 115.55(75.30,187.43) | 112.90(68.30,177.35) | Z=0.936 | 0.349 |
| PCT(ng/mL) | 9.98(2.09,41.09) | 9.45(1.42,44.12) | Z=0.351 | 0.726 |
| PT(s) | 15.20(13.50,17.30) | 14.40(12.70,16.90) | Z=2.610 | 0.009 |
| PT-INR | 1.35(1.21,1.52) | 1.30(1.18,1.50) | Z=2.014 | 0.044 |
| APTT(s) | 35.25(30.30,41.50) | 34.30(30.15,40.85) | Z=0.891 | 0.373 |
| FIB(g/L) | 5.39(3.60,10.29) | 5.50(3.75,12.50) | Z=0.723 | 0.470 |
| TT(s) | 15.70(11.92,17.12) | 15.50(5.33,17.30) | Z=0.967 | 0.334 |
| WBC(10^9^/L) | 12.00(7.27,17.75) | 11.77(7.41,17.80) | Z=0.063 | 0.950 |
| NEU(10^9^/L) | 10.69(6.09,16.15) | 10.16(6.30,16.45) | Z=0.023 | 0.981 |
| LYM(10^9^/L) | 0.66(0.38,1.03) | 0.60(0.35,0.99) | Z=1.500 | 0.134 |
| MON(10^9^/L) | 0.40(0.19,0.65) | 0.35(0.17,0.64) | Z=1.096 | 0.273 |
| Hb(g/L) | 105.00(88.00,126.00) | 105.00(90.00,126.00) | Z=0.410 | 0.682 |
| RDW(fL) | 48.35(45.00,54.23) | 48.90(45.10,53.95) | Z=0.272 | 0.786 |
| PLT(10^9^/L) | 140.50(90.00,224.50) | 140.00(76.00,222.50) | Z=0.554 | 0.580 |
| PDW | 14.85(12.30,16.60) | 15.00(11.90,16.60) | Z=0.417 | 0.677 |
| Plateletcrit | 0.16(0.10,0.24) | 0.16(0.09,0.24) | Z=0.771 | 0.441 |
| P-LCR | 11.20(10.10,12.20) | 11.20(10.10,12.05) | Z=0.644 | 0.520 |
| MPV(fL) | 34.30(26.70,41.90) | 34.20(26.50,40.55) | Z=0.327 | 0.744 |
| Interventions, n(%) |  |  |  |  |
| Deep venous catheterization | 397(78.15%) | 270(79.65%) | χ2=0.272 | 0.602 |
| Anticoagulant drugs | 258(50.79%) | 152(44.84%) | χ2=2.882 | 0.090 |
| Mechanical ventilation | 447(87.99%) | 302(89.09%) | χ2=0.238 | 0.626 |
| Hormones | 380(74.80%) | 242(71.39%) | χ2=1.217 | 0.270 |
| Vasoactive drugs | 480(94.49%) | 326(96.17%) | χ2=1.241 | 0.265 |
| CRRT | 260(51.18%) | 193(56.93%) | χ2=2.703 | 0.100 |
| Platelet transfusion | 77(15.16%) | 66(19.47%) | χ2=2.693 | 0.101 |
| Red blood cell transfusion | 210(41.34%) | 168(49.56%) | χ2=5.558 | 0.018 |
| Plasma infusion | 229(45.08%) | 156(46.02%) | χ2=0.072 | 0.788 |
| Infusion of cold precipitated clotting factors | 42(8.27%) | 47(13.86%) | χ2=6.772 | 0.009 |
| Infusion of human albumin | 366(72.05%) | 258(76.11%) | χ2=1.727 | 0.189 |
| Hospitalization Status |  |  |  |  |
| ICU Stay(Days) | 6.00(3.00,14.00) | 7.00(4.00,14.00) | Z=1.463 | 0.142 |
| Length of hospital stay(Days) | 16.00(8.00,28.00) | 17.00(9.00,28.00) | Z=0.723 | 0.470 |
| Z: Mann-Whitney test, χ²: Chi-square test | | | | |
| M: Median, Q₁: 1st Quartile, Q₃: 3st Quartile | | | | |
| APACHE II: acute physiology and chronic health evaluation II; SOFA: sequential organ failure assessment; HR: heart rate; SBP: systolic blood pressure; DBP: diastolic blood pressure; MAP: mean arterial pressure; RR: respiratory rate; AB, actual bicarbonate; OI, oxygenation index; TP: total protein; ALB: albumin; TBIL: total bilirubin; DBIL: direct bilirubin; ALT: alanine aminotransferase; AST: aspartate aminotransferase; K: potassium; Na: sodium; Cl: chloride; Ca: calcium; PO_4_: phosphorus; Mg: magnesium; BUN: blood urea nitrogen; Cr: creatinine; PCT: procalcitonin; PT: prothrombin time; PT-INR: prothrombin time-international normalisation ratio; APTT: activated partial thromboplastin time; FIB: fibrinogen; TT: thrombin time, WBC: white blood cells; NEU: neutrophils; LYM: lymphocytes; MON: monocytes; Hb: hemoglobin; RDW: red blood cell distribution width; PLT: platelet; PDW: platelet distribution width; P-LCR: platelet large cell ratio; MPV: mean platelet volume; CRRT: continuous renal replacement therapy; ICU: intensive care unit. | | | | |

Table S2. Baseline characteristics of candidate predictors in the validation set

| Variables | SALI (n=183) | Non-SALI (n=241) | Z/χ² | *P* |
| --- | --- | --- | --- | --- |
| Age, SD | 73.00(65.00,80.50) | 75.00(68.00,82.00) | Z=1.437 | 0.151 |
| DBIL, M (Q₁, Q₃) | 12.20(4.90,26.11) | 4.60(2.79,7.20) | Z=9.154 | <0.001 |
| AST, M (Q₁, Q₃) | 62.70(35.40,177.55) | 29.30(20.70,47.00) | Z=8.429 | <0.001 |
| PT-INR, M (Q₁, Q₃) | 1.56(1.44,1.74) | 1.26(1.14,1.38) | Z=12.547 | <0.001 |
| Vasoactive drugs, n(%) | 150(81.97%) | 185(76.76%) | χ2=1.698 | 0.193 |
| DBIL: direct bilirubin; AST: aspartate aminotransferase; PT-INR: prothrombin time-international normalisation ratio. | | | | |

Table S3. Definitions, units, coding, and assessment windows of predictors included in the final prediction model

| Predictor | Definition | Type | Unit/coding | Model specification | Assessment window |
| --- | --- | --- | --- | --- | --- |
| Age | Age at the index time (T0) | Continuous | Years | Entered on the original continuous scale; effect estimated per 1-year increase | T0 |
| DBIL | Earliest DBIL measurement obtained within the predictor-assessment window | Continuous | μmol/L | Entered on the original continuous scale; effect estimated per 1 μmol/L increase | From T0 to the earlier of 24 h after T0 or immediately before SALI onset |
| AST | Earliest AST measurement obtained within the predictor-assessment window | Continuous | U/L | Entered on the original continuous scale; effect estimated per 1 U/L increase | From T0 to the earlier of 24 h after T0 or immediately before SALI onset |
| PT-INR | Earliest PT-INR measurement obtained within the predictor-assessment window | Continuous | Unitless ratio | Entered on the original continuous scale; effect estimated per 1.0-unit increase | From T0 to the earlier of 24 h after T0 or immediately before SALI onset |
| Vasoactive-drug use | Administration of at least one of the following prespecified vasoactive agents within the predictor-assessment window: Norepinephrine; Epinephrine; Dopamine; Vasopressin; Phenylephrine; Dobutamine; Other vasopressor or inotropic agents. | Binary | 0 = no; 1 = yes | Entered as a binary indicator without transformation | From T0 to the earlier of 24 h after T0 or immediately before SALI onset |
| DBIL, direct bilirubin; AST, aspartate aminotransferase; PT-INR, prothrombin time–international normalized ratio; SALI, sepsis-associated liver injury; T0, the index time. Continuous predictors were entered into the final logistic regression model on their original measurement scales without categorization or transformation. Vasoactive-drug use was coded as 0 for no and 1 for yes. For patients who developed SALI within 24 h after T0, the predictor-assessment window was truncated immediately before the first laboratory result fulfilling the SALI criteria. Measurements and treatment information recorded at or after SALI onset were not eligible for predictor ascertainment. | | | | | |

Table S4. Comparison of the nomogram with the SOFA score benchmark

| Dataset | Nomogram AUC (95% CI) | SOFA AUC (95% CI) | ΔAUC | DeLong P | IDI (95% CI) |
| --- | --- | --- | --- | --- | --- |
| Training set | 0.925 (0.901–0.948) | 0.544 (0.492-0.597) | 0.381 | <0.001 | 0.553 (0.506-0.595) |
| Testing set | 0.918 (0.888–0.948) | 0.553 (0.490-0.614) | 0.365 | <0.001 | 0.576 (0.512-0.633) |
| AUC: area under the receiver operating characteristic curve; CI: confidence interval; SOFA: Sequential Organ Failure Assessment; IDI: Incremental discrimination improvement. Comparison of discriminative performance between the developed nomogram and the single-variable logistic model based on total SOFA score in the training and testing cohorts. | | | | | |

**Supplementary Figure**

**Figure S1A. Receiver operating characteristic curve of the nomogram in the testing cohort.** The AUC was 0.918 (95% CI, 0.888–0.948).

**Figure S1B. Receiver operating characteristic curve of the nomogram in the external geographic validation cohort.** The AUC was 0.884 (95% CI, 0.849–0.918).

**Figure S2A.** Calibration curve of the nomogram model in the testing set.

**Figure S2B.** Calibration curve of the nomogram model in the validation set.

**Figure S3A. Decision curve analysis of the nomogram in the testing cohort.** Although the full mathematical threshold range is displayed, clinical interpretation was restricted to the illustrative range of 0.10–0.40.

**Figure S3B. Decision curve analysis of the nomogram in the external geographic validation cohort.** Although the full mathematical threshold range is displayed, clinical interpretation was restricted to the illustrative range of 0.10–0.40.

**Figure S4.** **Temporal framework for predictor ascertainment and SALI outcome assessment.** T0 was defined as the index time at which a patient first fulfilled the Sepsis-3 criteria. SALI ascertainment began after T0 and continued until the first laboratory result fulfilling the prespecified SALI criteria or hospital discharge. The predictor-assessment window extended from T0, inclusive, to the earlier of 24 h after T0 or SALI onset, exclusive. The earliest eligible laboratory and vital-sign measurements within this window were used. Vasoactive-drug use was coded as present when at least one vasoactive agent was administered within the same window. Measurements and treatment information recorded at or after SALI onset were excluded from predictor ascertainment. (A) For patients who developed SALI within 24 h after T0, the predictor-assessment window was truncated immediately before SALI onset. (B) For patients who developed SALI after 24 h or remained free of SALI, the full 24-h predictor-assessment window was used; patients without SALI were followed until hospital discharge.

**Figure S5. Restricted cubic spline analyses of age, DBIL, AST, and PT-INR in the training cohort.** Restricted cubic splines with three knots placed at the 10th, 50th, and 90th percentiles were used to evaluate potential nonlinear associations between the continuous predictors and subsequent in-hospital SALI. (A) Age; (B) DBIL; (C) AST; and (D) PT-INR. No statistically significant evidence of nonlinearity was identified for age (P for nonlinearity = 0.572), DBIL (P for nonlinearity = 0.498), AST (P for nonlinearity = 0.062), or PT-INR (P for nonlinearity = 0.172).
